# Plain Water Intake, Sleep Quality, and Hydration Status of Pregnant Woman in Hainan, China: A Cross-Sectional Study

**DOI:** 10.3390/nu16111626

**Published:** 2024-05-26

**Authors:** Guotian Lin, Na Zhang, Zhixiong Lin, Limin He, Fan Zhang

**Affiliations:** 1 School of Public Health, Hainan Medical University, 3 Xue Yuan Road, Longhua District, Haikou 571199, China; linguotian123@163.com (G.L.); helimin@hainmc.edu.cn (L.H.); 2 School of Health Medicine, University of Sanya, 191 Xue Yuan Road, Jiyang District, Sanya 572022, China; 3 Department of Nutrition and Food Hygiene, School of Public Health, Peking University, 38 Xue Yuan Road, Haidian District, Beijing 100191, China; zhangna@bjmu.edu.cn; 4 Laboratory of Toxicological Research and Risk Assessment for Food Safety, Peking University, 38 Xue Yuan Road, Haidian District, Beijing 100191, China; 5 Department of Pediatric Internal Medicine, Haikou Hospital of The Maternal and Child Health, 6 Wen Tan Road, Guo Xing Avenue, Qiongshan District, Haikou 570203, China; zgyglzx@163.com; 6 Key Laboratory of Tropical Translational Medicine of Ministry of Educaiton, Hainan Medical University, 3 Xue Yuan Road, Longhua District, Haikou 571199, China

**Keywords:** hydration status, water intake, pregnant women

## Abstract

Objective: Both short and long sleep durations are associated with decreased kidney function; however, few studies have examined the relationship between sleep, hydration status, and plain water intake. This study aimed to assess the relationship between sleep quality, hydration status, and plain water intake in pregnant women. Methods: A cross-sectional study method was used to collect data from 380 pregnant women with regular examinations at the hospital between May 2019 and February 2021. Results: There were statistically significant differences in daily plain water intake (*x*^2^ = 14.118, *p* = 0.001), PSQI score (*x*^2^ = 77.708, *p* < 0.001), sleep duration (*x*^2^ = 67.569, *p* > 0.001), subjective sleep quality (*x*^2^ = 67.441, *p* = 0.001), time to fall asleep (*x*^2^ = 64.782, *p* < 001), sleep disorders (*x*^2^ = 70.853, *p* < 0.001), and daytime dysfunction (*x*^2^ = 38.441, *p* < 0.001) among different hydration status groups. Ordinal logistic regression results indicated that the intake of plain water ≥1500 mL/d (*OR* = 0.40, 95% *CI* = 0.24~0.67), good subjective sleep quality (*OR* = 0.15, 95% *CI* = 0.07~0.32), short time to fall asleep (*OR* = 0.32, 95% *CI* = 0.14~0.70), 8 h of sleep (*OR* = 0.06, 95% *CI* = 0.02~0.17), 6–7 h of sleep (*OR* = 0.19, 95% *CI* = 0.07~0.54), no sleep disturbance (*OR =* 0.31, 95% *CI* = 0.11~0.89), and high sleep efficiency (*OR* = 0.46, 95% *CI* = 0.03~0.79) were factors that were correlated with optimal hydration status. Sleep duration and daytime dysfunction partially mediated the effect of plain water intake on hydration status. The mediating effect of sleep duration was −0.036, accounting for 14.006% of the overall effect. The mediating effect of daytime dysfunction was −0.024, accounting for 9.459% of the overall effect. Conclusion: The hydration status in pregnant women may be affected by daily plain water intake and sleep quality.

## 1. Introduction

Water is a medium, raw material, and product for biochemical reactions in the body. It maintains normal osmotic pressure and electrolyte balance in body fluids, transfers the heat generated by energy metabolism to the skin through body fluids, and regulates body temperature through evaporation or sweat. Water also lubricates organs, joints, muscles, and tissues [1]. Under normal circumstances, water maintains a dynamic balance in the body, meaning the amount consumed and discharged is roughly equal. However, excessive or insufficient water intake affects this balance, changing the hydration status and impacting health. Excessive intake can lead to acute water poisoning and hyponatremia [2], while insufficient intake can cause dehydration, reducing cognitive [3,4,5,6] and physical abilities [4], and increasing the risk of urinary system diseases [7] and cardiovascular diseases [8]. Therefore, sufficient water intake is significant for health.

Sleep quality leads to changes in physiological function. Lack of sleep is closely correlated with diabetes, hypertension, obesity, and chronic kidney disease, impacting maternal and infant health [9,10,11]. Research shows that over 60% of pregnant women experience sleep problems during pregnancy, including difficulty falling asleep and frequently waking up at night [2,12]. Lack of sleep not only affects the mental state of expectant mothers but may also have a profound impact on the health of the fetus. Lack of sleep may lead to elevated blood pressure and emotional fluctuations, and even increase the risk of premature birth and low birth weight in pregnant women [13]. Recent studies have found that adults sleeping no more than six hours had a higher urine concentration than those sleeping about eight hours. In China, adults sleeping ≤6 h had a 42% increased risk of dehydration, compared to a 59% increase in the United States. It was also noted that no significant change in hydration status occurred when adult sleep duration reached or exceeded nine hours [14]. However, these studies focused only on adults, excluding pregnant women [15]. Research has shown that pregnant women are prone to sleep disorders, such as difficulty falling asleep, frequent waking, and insufficient sleep durations [16]. This phenomenon occurs in up to 63.80% of pregnant women during pregnancy, making it common [16]

The most abundant nutrient in the human body is water, accounting for about 50%~70% of its composition. It participates in the entire process of human metabolism and plays a crucial role in growth and development [10]. Pregnant women require more nutrients than non-pregnant women to meet their own and the fetuses’ needs [17]. Many nutrients in the body require water as a carrier. Moreover, energy consumption in adults is accompanied by water consumption: for every 4184 KJ of energy consumed, approximately 1 L of water is used. Pregnant women, especially in their second trimester and beyond, need more energy and consequently more water. Thus, maintaining an adequate hydration status during pregnancy is more important than before pregnancy for maternal and children’s health outcomes [17,18]. Hydrostasis in the human body refers to the fact that water intake and excretion remain roughly the same; no matter which side is more or less, it will break the water dynamic balance in the body and affect the change in hydration status. Some studies suggest that there may be a relationship between the regulation of hydration status and the occurrence and development of gestational hypertension and eclampsia [19]. In addition, studies have shown that low water intake may be one of the main mechanisms of diabetes development [20], thus it is speculated that the amount and type of water intake during pregnancy may also affect the risk of gestational diabetes.

Therefore, we hypothesized that the intake of plain water will affect hydration status and sleep quality, and sleep quality may be a mediating factor affecting the hydration status in pregnant women. We used urine osmotic pressure to investigate the correlation between plain water intake, sleep quality, and hydration status in pregnant women in Haikou, Hainan, China, and provide a scientific basis for maintaining adequate hydration status in pregnant women.

## 2. Research Objects and Methods

### 2.1. Participants and Study Design

Cross-sectional and convenience sampling methods were applied to collect data from 380 pregnant women at a cooperative hospital in Haikou, Hainan province, China, between May 2019 and February 2021. The study covered the following topics: (1) a structured questionnaire to collect age, gestational stage, occupation, and other demographic information; (2) a 24-h fluid intake questionnaire for 7 consecutive days to survey fluid intake behaviors; (3) the Pittsburgh Sleep Quality Index (PSQI) scale to assess sleep quality; (4) anthropometric measurement of Body Mass Index (BMI) to assess nutritional status; (5) and laboratory testing of urine indicators to determine hydration status.

Inclusion criteria were as follows: (1) age 21~35; (2) first pregnancy and singleton pregnancy; and (3) regular prenatal examination in Haikou Maternal and Child Health Hospital. Exclusion criteria were as follows: (1) recognition of the use of cigarettes or illegal drugs before or during pregnancy; (2) habitual drinking (the amount of ethanol consumption from alcohol drinking was >20 g/day) [21]; (3) high-intensity sports activities; (4) communication barriers; (5) oral diseases, endocrine diseases, urinary diseases, digestive diseases, cardiovascular diseases or mental disorders; and (6) diabetes or other diseases before pregnancy [21].

### 2.2. Sample Size Calculation

This study used the formula $N=\frac{t^{2}P(1-P)}{e^{2}}$, with a 95% confidence level, which means *t* = 1.96; According to published literature, the incidence of suitable hydration status in the population is 9.8% [22], which is *p* = 0.0988. The sampling error range did not exceed ±3%, thus *e* = 3% and *N* = 377.313, giving an integer of 380.

### 2.3. Ethical Standards

This research protocol was approved by the Ethics Review Committee of Hainan Medical University, with an ethics approval project identification code of 2018-4. The approval date of the ethical statement was 27 December 2018. The research protocol was registered on the website of the China Clinical Trial Registration Center, with trial registration number Chi CTR800019284. The study was conducted in accordance with the principles of the Helsinki Declaration, and all participants voluntarily read and signed two copies of the informed consent form before the start of the study.

### 2.4. Fluid Intake Behaviors

A 24-h fluid intake questionnaire was administered for 7 consecutive days, where participants estimated the amount and types of fluid intake, such as ordinary water (e.g., plain water, bottled water), sugary drinks, milk, and dairy products, and the quantities of each intake using a scaled cup. The scale of the cup was accurate to 10 mL. The recommended fluid intake values were 1500~1700 mL for the first trimester, 1700~1900 mL for the second and the third trimesters as per the Chinese Dietary Nutrient Reference Intake (2013), which was used to evaluate the water intake status of pregnant women. Eight periods of plain water intake were recorded as: before breakfast, at breakfast, between breakfast and lunch, at lunch, between lunch and dinner, at dinner, after dinner, and at nighttime.

### 2.5. Sleep Quality Assessment

The Pittsburgh Sleep Quality Index (PSQI) is a commonly used sleep quality assessment tool developed by Buysse in 1989. This scale is based on the principle of multidimensional evaluation and scores seven different aspects of sleep quality, including subjective sleep quality, sleep duration, time to fall asleep, sleep efficiency, sleep disorders, hypnotic drug use, and daytime dysfunction. The total score was obtained by adding up the scores for each aspect, and a higher total score indicates poorer sleep quality [23]. Each dimension was assigned 0–3 points according to the 4-level Likert method; the scoring requirements for each dimension were as follows:

(1) Subjective sleep quality: According to the response score in item 6, good, poor, and very poor were scored as 1, 2, and 3 points, respectively. A score of 1 indicated good sleep quality, while a score of 2–3 indicated poor sleep quality.
(2) Time to fall asleep: According to the cumulative scores of items 2 and 5a, the cumulative scores were 0 points, 1~2 points, 3~4 points, and 5~6 points, respectively, counted as 0, 1, 2, and 3 points. A score of 0–1 indicated a short time, and a score of 2–3 indicated a long time.
(3) Sleep duration: >7 h, 6~7 h, 5~6 h, and <5 h were counted as 0, 1, 2, and 3 points, respectively.
(4) Sleep efficiency: Sleep efficiency = (Sleep duration/Bed duration) × 100%, where Bed duration = Wake up time − Bed time. Sleep efficiency > 85%, 75%~84%, 65%~74%, and <65% were counted as 0, 1, 2, and 3 points, respectively.
(5) Sleep disturbance: According to the cumulative scores of items 5b–5j, the cumulative scores of 0, 1~9, 10–18, and 19~27 were counted as 0, 1, 2, and 3 points, respectively. A score of 0 or 1 indicated the absence of sleep disorders, while a score of 2 or 3 indicated the presence of sleep disorders.
(6) Hypnotic drugs: According to the response score in item 7, medication frequency of 0, <1 time/week, 1~2 times/week, and ≥3 times/week were counted as 0, 1, 2, and 3 points, respectively.
(7) Daytime dysfunction: According to the cumulative scores of items 8 and 9, the cumulative scores of 0, 1~2, 3~4, and 5~6 were counted as 0, 1, 2, and 3 points, respectively. A point of 0 or 1 indicated the absence of daytime dysfunction, while a point of 2 or 3 indicated the presence of daytime dysfunction.

The total PSQI score was the sum of all the points from the various dimensions. Sleep quality was divided into four degrees: good (0~5 points), decent (6~10 points), average (11~15 points), and very poor (16~21 points).

### 2.6. Demographic Factors

#### 2.6.1. Occupation

According to the occupational classification standards [24], occupation can be divided into four groups: (1) unemployed, which refers to people who have not engaged in any occupation or have no fixed occupation; (2) freelance, which refers to a profession that breaks free from the jurisdiction of enterprises and companies, manages itself, and focuses on individual labor; (3) blue collar, an occupation that primarily relies on physical strength to complete work tasks. This type of occupation usually requires a lot of physical labor, for example, construction workers, farmers, porters, salespeople, etc.; and (4) white-collar: an occupation that primarily relies on intelligence, knowledge, and professional skills to complete work tasks. This type of occupation typically requires a higher educational background and professional skills, for example, scientists, doctors, teachers, lawyers, etc. Occupations other than the aforementioned unemployed, freelance, blue-collar, and white-collar were classified as “Others”.

#### 2.6.2. Determination of Pregnancy

The period from conception to 12 weeks of pregnancy is called the first trimester, the period between 13 and 28 weeks after pregnancy is called the second trimester, and the period from 29 weeks after pregnancy to full-term delivery is called the third trimester [22].

#### 2.6.3. Anthropometric Measurements

The day participants were included in the study during their antenatal care visits, their body heights and weights were measured using an automatic anthropometer (HDM-300; Chinese, Yiwu, China). The measurement accuracy of this instrument was ±0.1 cm for height and 0.1 kg for weight. When measuring heights and weights, the trained nurses required participants to put down all belongings, take off their shoes, socks, and hats, and stand with their feet in the center of the instrument’s bottom plate with legs straight, toes separated by about 60 degrees, the torso naturally straightened, the upper limbs naturally drooped, their backs facing the pillars, their heads straightened, maintaining a stable body, and looking straight ahead in a “three point line” standing posture. The nutritional status was assessed as $BMI=\frac{Weight\;(kg)}{{Height}^{2}\;(m^{2})}$. BMI < 18.5 kg/m^2^ indicated underweight, BMI between 18.5 kg/m^2^ and 23.9 kg/m^2^ indicated normal weight, BMI between 24 kg/m^2^ and 27.9 kg/m^2^ indicated overweight, and BMI ≥ 28 kg/m^2^ indicated obesity [22].

### 2.7. Urine Osmolality

The first morning urine was collected in a dark container and its osmolality was measured using an osmolality meter (SMC 30C; Tianhe; Tianjin, China) following the standard operating procedure of the Chinese Standard Practice for Drug Inspection, 2016.

### 2.8. Hydration Status

The hydration status was categorized, based on urine osmotic pressure values, into optimal hydrated status (≤0.500 Osm/kg), normal hydrated status (0.501–0.799 Osm/kg), and dehydrated status (≥0.800 Osm/kg) [21]

### 2.9. Temperature and Humidity in the Environment

Daily meteorological data, including temperature, relative humidity, sunshine hours, and air pressure from May 2019 to February 2021 were obtained from the China Meteorological Network, and the observed months were divided into May–July (high-temperature months) and August–April of the following year (non-high temperature months). During the study period, the average temperature in Haikou, Hainan Province, was 27.6 ± 3.3 °C, the average relative humidity was 78.3 ± 7.9%, the average hours of sunshine were 6.3 ± 3.7 h, and the average air pressure was 100.1 ± 0.5 KPa.

### 2.10. On-Site Investigation and Quality Control

Prior to the project’s commencement, the project team prepared a standard operating procedure (SOP) manual for investigating pregnant women’s hydration status and conducted unified training for the investigators. The project team leader introduced and explained the research process and specific details to all investigators and trained them on how to fill in the questionnaire and use the water cups. A post-training test was conducted and the ones passing the test were permitted to conduct the on-site investigations. The project team leader supervised the entire testing process and addressed and corrected problems promptly. Throughout the study, investigators maintained daily telephone contact with the participants, collected drinking water questionnaires as per the study plan, verified data on daily fluid intake and types, checked for suspicious data on-site, and corrected any errors.

### 2.11. Statistical Analysis

Data were input and verified in Excel 2016; statistical analyses were then conducted using SPSS 26.0. When the data distribution was normal, the concentration and dispersion trends were described using mean ± SD. When the data distribution was skewed, *M* (*QR*) was used to describe the concentration and dispersion trends. Differences in hydration status among different ethnic groups, ages, gestational stages, maternal BMIs, occupations, and sleep qualities were compared using the Chi-square test. Hydration status was set as the dependent variable, and sleep duration, sleep quality, time to fall asleep, sleep disorders, and daytime dysfunction were set as independent variables, and ordered logistic regression analysis was conducted. Hydration status was set as the dependent variable, various dimensions of sleep quality and plain water intake were set as independent variables, and linear regression analysis was used to obtain the interactive effects of the independent variables on hydration status. The Bootstrap method was used to verify the mediating effect of sleep quality on hydration status and plain water intake. *p* < 0.05 indicated a statistically significant difference.

## 3. Results

### 3.1. Plain Water Intake by Pregnant Women

As Table 1 shows, the daily plain water intakes of the participants increased as their gestational weeks increased, and a significant statistical difference was found (*p* < 0.001). In the first trimester, 3.5% of participants (5/142) consumed the recommended water intake. In the second and third trimesters, the proportions meeting the recommended intake were 0.05% (1/184) and 5.5% (3/54), respectively. The normal BMI group had significantly higher daily plain water intake than the other BMI groups (*p* < 0.01). No significant difference was found within different ethnic, age, or occupation groups (*p >* 0.05).

### 3.2. Plain Water Intake in Pregnant Women over Eight Time Periods

As Table 2 shows, among the 380 pregnant women, the proportion of plain water intake ≥1500 mL/d was 21.6% (82/380). There were statistically significant differences in total plain water intake between different time periods (*H* = 1630.6, *p* < 0.001), at dinner (215.7 mL) > between lunch and dinner (214.3 mL) > at lunch (210.7 mL) > between breakfast and lunch (202.1 mL) > after dinner (201.4 mL) > at breakfast (125.7 mL) > nighttime (119.3 mL) > before breakfast (44.3 mL). There were statistically significant differences between the two groups at lunch and at nighttime. The ≥1500 mL/d group consumed more plain water at lunchtime than the <1500 mL/d group; however, the values were reversed at nighttime.

### 3.3. Univariate Analysis of Hydration Status in Pregnant Women

There were significant differences in daily plain water intake (*x*^2^ = 14.118) and total PSQI scores (*x*^2^ = 77.708) among different trimester groups in different hydration statuses (*p* < 0.001). A higher proportion of pregnant women with daily plain water intake ≥1500 mL/d (30.4%) and total PSQI scores, indicating good sleep quality (89.2%), were in the optimal hydration status group compared to the other two groups. No other differences were statistically significant (all *p* > 0.05). See Table 3.

### 3.4. Univariate Analysis between Hydration Status and Multiple Dimensions of Sleep Quality among Pregnant Women

Statistically significant differences were found in sleep duration (*x*^2^ = 67.569), sleep quality (*x*^2^ = 67.441), time to fall asleep (*x*^2^ = 64.782), sleep disturbance (*x*^2^ = 70.853), and daytime dysfunction (*x*^2^ = 38.441) among different hydration status groups (*p* < 0.001). The optimal hydration status group had the highest rates of 8 h of sleep (87.3%), good sleep quality (96.1%), short time to fall asleep (98.0%), absence of sleep disturbance (98.0%), and absence of daytime dysfunction (86.3%) compared with the other two groups. However, the differences in sleep efficiency between the different hydration status groups were not statistically significant (*x*^2^ = 1.252, *p*> 0.05), as shown in Table 3.

### 3.5. Univariate Analysis between Level of Plain Water Intake and Multiple Dimensions of Sleep Quality among 380 Pregnant Women

Statistically significant differences were found in sleep duration (*x*^2^ = 6.638) and daytime dysfunction (*x*^2^ = 5.441) among the two groups in the level of plain water intake (*p* < 0.05). Pregnant women with intake ≥1500 mL/d had significantly higher rates of 8 h of sleep (73.2%) and no daytime dysfunction (87.8%) compared with those who consumed <1500 mL, as shown in Table 4.

### 3.6. Multivariate Ordered Logistic Regression Analysis of Hydration Status among Pregnant Women

An ordinal logistic regression model was established, with the hydration status of 380 pregnant women as the dependent variable, and sleep duration, sleep quality, time to fall asleep, sleep disturbance, daytime dysfunction, sleep efficiency, and daily plain water intake as independent variables. Specific assignments are detailed in Table 5.

The parallel line test for this model was *x*^2^ = 17.734 and *p* = 0.071, indicating that the proportional advantage hypothesis exists. Model fit information was *x*^2^ = 85.285, *p* < 0.001, suggesting a good model fit. In the ordered logistic regression analysis, pregnant women with good sleep quality were 0.15 times more likely to experience dehydration than those with poor sleep quality (*OR* = 0.15, 95% *CI:* 0.07~0.32). The probability of dehydration in women who took a short time to fall asleep was 0.32 times higher than in women who took a long time to fall asleep (*OR* = 0.32, 95% *CI:* 0.14~0.70). Taking sleep time < 6 h as a reference, women whose sleep duration ≥ 8 h were 0.06 times more likely to experience dehydration (*OR* = 0.06, 95% *CI:* 0.02~0.17), and women whose sleep duration was 6–7 h were 0.19 times more likely to experience dehydration (*OR* = 0.19.95% *CI:* 0.07~0.54). The probability of dehydration in individuals with no sleep disturbance was 0.31 times higher than in those with sleep disturbance (*OR* = 0.31, 95% *CI:* 0.11~0.89), and the probability of dehydration in those with high sleep efficiency was 0.46 times higher than in those with low sleep efficiency (*OR* = 0.046, 95% *CI:* 0.03~0.79). Specific assignments are detailed in Table 6.

### 3.7. Analysis of the Regulatory and Mediating Effects of Hydration Status on Water Intake and Sleep Quality

The hydration status was set as the dependent variable, and various dimensions of sleep quality and plain water intake were set as independent variables. The results showed that there was no interaction between plain water intake and the various dimensions of sleep quality (*p* > 0.05), as shown in Table 7.

**Figure 1:**
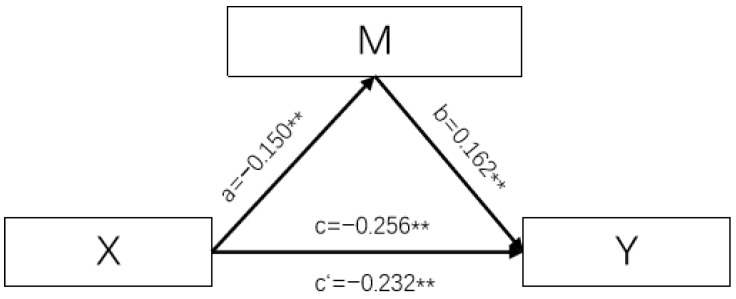
Pathmap showing plain water intake ≥ daytime dysfunction ≥ hydration status. (Notes: X is plain water intake, M is daytime dysfunction, and Y is hydration status. The values corresponding to a, b, c, and c′ are the values of the regression coefficients *β*, ** *p* < 0.001).

**Figure 2:**
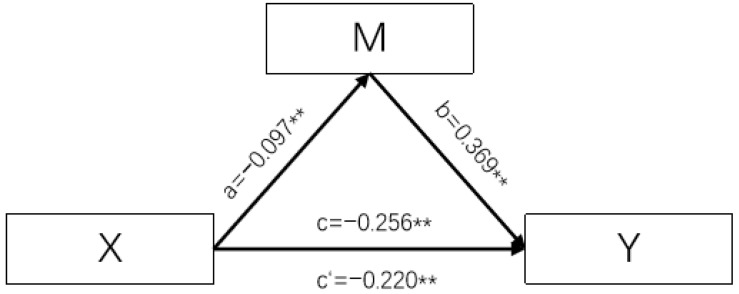
Pathmap showing plain water intake ≥ sleep duration ≥ hydration status. (Notes: X is plain water intake, M is Sleep duration, and Y is hydration status. The values corresponding to a, b, c, and c′ are the values of the regression coefficients *β*, ** *p* < 0.001).

The Bootstrap method was used to analyze the mediating effects of various dimensions of sleep quality on water intake and hydration status, with a self-sampling size of 5000. The results showed that sleep duration and daytime dysfunction partially mediate the relationship between water intake and hydration status. The mediating effect of sleep time was −0.036, accounting for 14.01% of the overall effect and the mediating effect of daytime dysfunction score was −0.024, accounting for 9.46% of the overall effect. The results are shown in Table 8 and Figure 1 and Figure 2.

## 4. Discussion

### 4.1. Pregnant Women Consume Insufficient Amounts of Drinking Water during Different Pregnancy Periods

The percentages of pregnant women achieving the recommended drinking water intake volumes in the first, second, and third trimesters in China were 3.50%, 0.05%, and 5.50%, respectively. The daily plain water intake volume found in this study (1230 ± 10 mL) was lower than the daily average of 98 pregnant women (1279.85 ± 670.28 mL) in a study by Zhang Liyan [23]. Additionally, the proportion of recommended intake reached in this study (3.50%) was lower than the 36.7% reported by Zhang Liyan (36/96) [23]. This discrepancy might be due to different investigative methods: Zhang Liyan [23] utilized the 24-h dietary review method and the food frequency method, which do not consider other influencing factors and may have recall bias.

Our study, along with the study by Zhang Liyan et al. [23], found that the average fluid intake of pregnant women was higher than that of college students (1135 mL) [25], suggesting pregnant women’s water intake is higher than that of the general population and increases with gestational age. This could be linked to specific physiological changes during pregnancy. For instance, the incidence of dry mouth in the second and third trimesters was 32.8% [26], potentially leading to an increased demand for water by pregnant women. Compared with international studies on pregnant women’s water intake, a 1977–1978 study used a 2-day dietary record method to study 188 pregnant women, revealing an average intake of 2.1 L/d [27]. In 2016, Indian researchers conducted a 7-day 24-h drinking record study with 300 pregnant women, finding an average daily intake of 2.3 L/d, with about 42% not meeting the recommended intake of 2048 mL/d [22]. These figures are significantly higher than those found in China and in this study. The difference may be attributed to varying domestic and international dietary structures [21,28,29]. In China, diets are predominantly plant-based, including soup and porridge, whereas foreign diets lean more towards animal foods, and their cooking methods often result in greater water loss [21]. Additionally, pregnant women may lack knowledge about healthy water intake and exhibit unhealthy water consumption behaviors [30], often unaware of their own water loss and knowledge gaps. This could lead to lower water consumption and failure to meet recommended amounts.

This study revealed that 22.2%, 15.2%, and 18.3% of pregnant women were dehydrated in the first, second, and third trimesters, respectively. It also found that pregnant women with a plain water intake of ≥1500 mL/d were 0.40 times that of those with an intake <1500 mL/d (*OR* = 0.40, 95% *CI* 0.24–0.67). Thus, it was evident that hydration in the first trimester requires further improvement. Further research has shown that water intake varies across different trimesters. Therefore, water intake was likely a contributing factor to varying hydration statuses.

### 4.2. Pregnant Women with Poor Sleep Conditions Are More Prone to Dehydration

This study’s univariate analysis revealed a statistically significant difference between hydration status and PSQI score groups ($\chi^{2}$ = 77.708, *p* < 0.001), with 89.2% (91/102) of pregnant women with good sleep quality scoring higher than the other two groups. In the ordered logistic regression analysis, pregnant women with good sleep quality were 0.15 times more likely to experience dehydration than those with poor sleep quality (*OR* = 0.15, 95% *CI:* 0.07~0.32). The probability of dehydration in women who took a short time to fall asleep was 0.32 times higher than in women who took a long time to fall asleep (*OR* = 0.32, 95% *CI:* 0.14~0.70). Taking sleep time < 6 h as a reference, women whose sleep duration ≥ 8 h were 0.06 times more likely to experience dehydration (*OR* = 0.06, 95% *CI:* 0.02~0.17), and women whose sleep duration was 6–7 h were 0.19 times more likely to experience dehydration (*OR* = 0.19, 95% *CI:* 0.07~0.54). The probability of dehydration in individuals with no sleep disturbance was 0.31 times higher than in those with sleep disturbance (*OR* = 0.31, 95% *CI:* 0.11~0.89), and the probability of dehydration in those with high sleep efficiency was 0.46 times higher than in those with low sleep efficiency (*OR* = 0.046, 95% *CI:* 0.03~0.79). This indicates that pregnant women with poor sleep were more likely to experience dehydration, as sleep affects various physiological functions and diseases. Several studies have shown that sleep deprivation affects physiological processes such as metabolism and immunity [29]. Moreover, sleep disturbance has been identified as a risk factor for hypertension, central nervous system disease, and cardiovascular disease [30,31], and poor sleep quality has been linked to an increased risk of abnormal kidney function [32]. The body’s hydration status is determined by water consumption and discharge, regulated by factors including plasma osmolality, blood pressure, the nervous system, hormones, and renal function [21]. Sleep may influence the amount of water discharged by affecting these factors, thereby impacting hydration status in women during pregnancy. A key physiological pathway connecting hydration and sleep is the circadian release of antidiuretic hormone or vasopressin [33,34]. Vasopressin release increases later in sleep, helping to regulate hydration during sleep. This means that if late sleep is disrupted, the individual may not experience the increased vasopressin release required to promote water homeostasis in the body, which may increase the occurrence of dehydration.

### 4.3. Sleep Quality May Be a Mediating Variable between Plain Water Intake and Hydration Status

The results of the study showed that sleep duration and daytime dysfunction partially mediate the relationship between water intake and hydration status. In our study, it was also mentioned that the subjects drank less water at night than during other periods, which may be associated with physiological changes during pregnancy, in order to reduce the number of nighttime wake-ups and reduce the amount of water consumed at night while also ensuring adequate sleep time [16]. From our data, it is unclear what causes this link, i.e., whether shorter sleep duration affects antidiuretic hormone release, affecting kidney urine concentration. For example, short sleep duration may be associated with hydration-related behaviors, such as drinking fewer fluids. Although there is little literature on the adverse effects of sleep deprivation or sleep deprivation on hydration status and thirst [35], there have been reports of recurrent headaches and similar dehydration symptoms in sleep-deprived subjects.

## 5. Limitations

Currently, all data from our study are observational and come from cross-sectional studies, thus the associated results from this study should not be considered causal. In addition, it is not possible to investigate the direction of this association, and it is subject to reverse causality bias, i.e., the hydration status may affect sleep duration, although previous reports do not support this view. During the process of on-site implementation, the research subjects did not fill in the dietary intake, thus these data are missing. Future studies could test this hypothesis to further unravel the direction of this association. Finally, we have no information on vasopressin to investigate how differences in vasopressin may potentially modulate the relationship between sleep and hydration status.

## 6. Conclusions

The hydration status in pregnant women may be affected by daily plain water intake and sleep quality, and Chinese pregnant women do not consume sufficient water. Therefore, there is a beneficial aspect to the health of pregnant women in educating them about drinking sufficient quantities of water.

## Figures and Tables

**Table 1:** Plain water intake by pregnant women.

Variable	Class	Level of Plain Water Intake (mL)	*t*/*F*	*p*
Ethnic group	Han	1332 ± 9	−1.021	0.308
	non-Han	1349 ± 13		
Age (years)	22–29	1334 ± 11	−0.425	0.671
	30–34	1349 ± 10		
gestational stage	First trimester	1230 ± 10	96.95	<0.001
	Second trimester	1386 ± 9		
	Third trimester	1455 ± 17		
BMI (kg/m^2^)	Underweight	1307 ± 32	3.922	0.009
	Normal	1376 ± 25		
	Overweight	1361 ± 25		
	Obesity	1320 ± 10		
occupation	Unemployed	1312 ± 18	0.981	0.418
	Freelance	1351 ± 21		
	White collar	1352 ± 16		
	Blue collar	1335 ± 10		
	others	1261 ± 51		

(Note: The level of plain water intake is described in terms of mean ± SD).

**Table 2:** Plain water intake in pregnant women over eight time periods.

Eight Time Periods	Level of Plain Water Intake (M(QR), mL)		Total	*Z*	*p*
	≥1500 mL/d (*n* = 82)	<1500 mL/d (*n* = 298)			
before breakfast	43.6 (44.2)	48.6 (55.7)	44.3 (46.4)	−1.046	0.295
at breakfast	126.4 (60.4)	117.1 (74.7)	125.7 (87.9)	−1.083	0.279
Between breakfast and lunch	201.4 (64.3)	210.7 (61.8)	202.1 (65.4)	−1.171	0.242
at lunch	207.1 (68.6)	223.6 (59.2)	210.7 (58.6)	−2.746	0.006
Between lunch and dinner	207.9 (74.6)	230.0 (77.5)	214.3 (78.2)	−1.844	0.065
at dinner	214.2 (54.3)	219.3 (55.4)	215.7 (56.8)	−0.242	0.808
after dinner	199.3 (83.2)	202.1 (77.5)	201.4 (81.1)	−1.220	0.223
at nighttime	140.0 (60.0)	119.3 (55.7)	119.3 (61.5)	−2.872	<0.001

Note: M (QR) represents the median (quartile range).

**Table 3:** Univariate analysis of the hydration status of the pregnant women.

Variable	Class	Hydration Status [*n* (%)]			*x* ^2^	*p*
		Optimal Hydration Status (*n* = 102)	Normal Hydrated Status (*n* = 212)	Dehydrated Status (*n* = 66)		
Ethnic group	Han	68 (66.7)	145 (68.4)	43 (65.2)	0.272	0.873
	non-Han	34 (33.3)	67 (31.6)	23 (34.8)		
Age (years)	22–29	46 (45.1)	119 (56.1)	32 (48.5)	3.719	0.156
	30–34	56 (54.9)	93 (43.9)	34 (51.5)		
gestational stage	First trimester	40 (39.2)	76 (35.8)	26 (39.4)	4.141	0.387
	Second trimester	53 (52.0)	103 (48.6)	28 (42.4)		
	Third trimester	9 (8.8)	33 (15.6)	12 (18.2)		
BMI (kg/m^2^)	Underweight	8 (7.8)	11 (5.2)	6 (9.1)	5.819	0.444
	Normal	64 (62.7)	122 (57.5)	34 (51.5)		
	Overweight	23 (22.5)	58 (27.4)	16 (24.2)		
	Obesity	7 (7.0)	21 (9.9)	10 (15.2)		
occupation	Unemployed/housewives	16 (15.7)	38 (17.9)	7 (10.6)	6.016	0.645
	Freelance	11 (10.8)	33 (15.6)	12 (18.2)		
	White collar	31 (30.4)	47 (22.2)	17 (25.8)		
	Blue collar	43 (42.2)	92 (43.4)	30 (45.4)		
	others	1 (0.9)	2 (0.9)	0 (0.0)		
Plain water intake	≥1500 mL/d	31 (30.4)	47 (22.2)	4 (6.1)	14.118	0.001
	<1500 mL/d	71 (69.6)	165 (77.8)	62 (93.9)		
PSQI total points	Very good	91 (89.2)	160 (75.5)	19 (28.8)	77.708	<0.001
	Good	4 (3.9)	33 (15.6)	31 (47.0)		
	General	7 (6.9)	19 (8.9)	16 (24.2)		
Sleep duration	≥8 h	89 (87.3)	136 (64.2)	22 (33.3)	67.569	<0.001
	6~7 h	13 (12.7)	65 (30.7)	28 (42.4)		
	<6 h	0 (0.0)	11 (5.1)	16 (24.3)		
Sleep quality	Good	98 (96.1)	199 (93.9)	39 (59.1)	67.441	<0.001
	Poor	4 (3.9)	13 (6.1)	27 (40.9)		
Time to fall asleep	Time to fall asleep is short	100 (98.0)	188 (88.7)	36 (54.5)	64.782	<0.001
	Time to fall asleep is long	2 (2.0)	24 (11.3)	30 (45.5)		
Sleep efficiency	High	76 (74.5)	149 (70.3)	44 (66.7)	1.252	0.535
	Low	26 (25.5)	63 (29.7)	22 (33.3)		
Sleep disturbance	Yes	2 (2.0)	9 (4.2)	24 (36.4)	70.853	<0.001
	No	100 (98.0)	203 (95.8)	42 (63.6)		
daytime dysfunction	Yes	88 (86.3)	177 (83.5)	33 (50.0)	38.441	<0.001
	No		35 (16.5)	33 (50.0)		

**Table 4:** Univariate analysis of the level of plain water intake and multiple dimensions of sleep quality among pregnant women.

Variable	Class	Level of Plain Water Intake [*n* (%)]		*x* ^2^	*p*
		≥1500 mL/d (*n* = 82)	<1500 mL/d (*n* = 298)		
Sleep duration	≥8 h	60 (73.2)	187 (62.8)	6.638	0.041
	6~7 h	21 (25.6)	85 (28.5)		
	<6 h	1 (1.2)	26 (8.7)		
Sleep quality	Good	73 (89.0)	263 (88.3)	0.037	0.847
	Poor	9 (11.0)	35 (11.7)		
Time to fall asleep	Time to fall asleep is short	67 (73.2)	257 (79.3)	1.052	0.305
	Time to fall asleep is long	15 (26.8)	41 (20.7)		
Sleep efficiency	High	60 (74.1)	206 (69.6)	0.614	0.433
	Low	21 (25.9)	90 (30.4)		
Sleep disturbance	Yes	10 (12.2)	25 (8.4)	1.114	0.291
	No	72 (87.8)	273 (91.6)		
daytime dysfunction	Yes	10 (12.2)	72 (24.2)	5.441	0.020
	No	72 (87.8)	226 (75.8)		

**Table 5:** Assignment of the ordered logistic regression model.

Code	Variable	Assignment
Y	Hydration status	0 = optimal hydration status, 1 = normal hydration status, and 2 = dehydrated status
X1	Sleep duration	0 = ≥8 h, 1 = 6–7 h; 2 = <6 h
X2	Subjective sleep quality	0 = good, and 1 = poor
X3	Time to fall asleep	0 = short, 1 = long
X4	Sleep disturbance	0 = without sleep disturbance, 1 = with sleep disturbance
X5	Daytime dysfunction	0 = without daytime dysfunction, 1 = with daytime dysfunction
X6	Sleep efficiency	0 = high sleep efficiency; 1 = low sleep efficiency
X7	Daily plain water intake	0 = daily plain water intake ≥ 1500 mL/d; 1 = daily plain water intake < 1500 mL/d

**Table 6:** Multivariate ordered logistic regression analysis of hydration status in pregnant women.

	Variable	*β*	Standard Error	Wald	*p*	*OR*	*OR* 95% *CI*	
threshold	Normal hydration status	−6.577	0.787	69.795	-	-	-	-
	Dehydrated status	−3.022	0.706	18.311	-	-	-	-
position	Plain water intake ≥ 1500 mL/d	−0.922	0.266	12.032	0.001	0.40	0.24	0.67
	Good sleep quality	−1.924	0.399	23.192	<0.001	0.15	0.07	0.32
	Short time to fall asleep	−1.15	0.404	8.088	0.004	0.32	0.14	0.7
	Sleep duration ≥ 8 h	−2.875	0.557	26.682	<0.001	0.06	0.02	0.17
	Sleep duration for 6–7 h	−1.639	0.526	9.731	0.002	0.19	0.07	0.54
	Without Sleep disturbance	−1.178	0.539	4.775	0.029	0.31	0.11	0.89
	High sleep efficiency	−0.776	0.278	7.77	0.005	0.46	0.03	0.79

**Table 7:** The regulatory effects of various dimensions of plain water intake and sleep quality on hydration status.

Interaction Item	*β*	Standard Error	*t*	*p*
Plain water intake × Subjective sleep quality	0.017	0.034	0.484	0.629
Plain water intake × Time to fall asleep	−0.012	0.038	−0.323	0.747
Plain water intake × Sleep duration	0.057	0.045	1.267	0.206
Plain water intake × Sleep disturbance	0.039	0.047	0.835	0.404
Plain water intake × Daytime dysfunction	0.028	0.035	0.805	0.421

Note: ×: The interaction of two factors.

**Table 8:** Analysis of the mediating effect of sleep quality on plain water intake and hydration status.

Path Item	Test Conclusion	Total Effect	Mediating Effect	Direct Effect	Proportion of Effect	95% *CI*
Plain water intake → Daytime dysfunction → hydration status	Partial intermediaries	−0.256 **	−0.024	0.232 **	9.46%	−0.071~−0.011
Plain water intake → Sleep duration → hydration status	Partial intermediaries	−0.256 **	−0.036	−0.220 **	14.01%	−0.095~−0.016

Note: ** *p* < 0.01, →: the effect of the former on the latter.

## Data Availability

The data are available from the corresponding authors upon reasonable request. The data are not publicly available due to there is a need to protect patient privacy and ethics.
